# Bacteriophage therapy cures a recurrent *Enterococcus faecalis* infected total hip arthroplasty? A case report

**DOI:** 10.1080/17453674.2021.1968714

**Published:** 2021-08-20

**Authors:** Ann-Sophie Neuts, Hanneke J Berkhout, Anita Hartog, Jon H M Goosen

**Affiliations:** 1 Department of Orthopaedic Surgery, Sint Maartenskliniek, Nijmegen; 2 Department of Medical Microbiology and Infectious Diseases, Canisius-Wilhemina Hospital, Nijmegen; 3 NIZO, Ede; 4 Department of Orthopaedic Surgery, Sint Maartenskliniek, Nijmegen, The Netherlands

A 76-year-old male patient presented with osteoarthritis of the left hip. He had no relevant medical history and had been professionally active in agriculture. Cemented total hip arthroplasty (THA), performed in September 2015, was complicated by an extensive hematoma and wound leakage on the second postoperative day. Wound leakage persisted for another 8 days, and debridement was performed. All 6 tissue samples showed the presence of *Enterococcus faecalis*, and the patient was treated with intravenous teicoplanin 600 mg twice a day for 3 months. After this treatment, the infection recurred, and a 2-stage septic revision was scheduled. The prosthesis and all surrounding cement were removed in February 2016. All samples taken showed growth of *E. faecalis.* After susceptibility testing, teicoplanin was restarted and continued for 6 weeks. Reimplantation followed in May 2016 after an antibiotic-free period of more than 6 weeks. Tissue samples obtained at that time showed no bacterial growth. In December 2016, the patient complained of pain and discomfort. 2 separate joint aspirations showed no bacterial growth, but serial radiological images showed loosening of the femoral stem. The patient was planned for revision surgery in January 2017. In 5 out of 7 samples, *E. faecalis* was cultured. After susceptibility testing, teicoplanin was restarted for another 3 months at the same dose. In June 2017, an aspiration, performed due to persistent pain, turned out to be positive for *E. faecalis* again. Extraction of the hip prosthesis followed and teicoplanin was restarted for another 6 weeks. After an antibiotic-free period of 2 weeks, open biopsies were taken. They all showed no bacterial growth. 4 weeks later, the hip was reimplanted (Figure 1). At the time of reimplantation, *E. faecalis* was cultured in 4 out of 7 samples. Due to kidney failure during the last period of teicoplanin therapy, this antibiotic regime could not be restarted. Postoperatively, oral amoxicillin 1,000 mg was administered 4 times a day for 3 months. In the summer of 2018, a new sample was obtained because of pain. The known *E. faecalis* was again cultured. A Girdlestone procedure was not accepted by the patient, who opted for suppressive therapy with doxycycline 200 mg once a day. Due to gastrointestinal side effects, the doxycycline dose was reduced to 100 mg once a day in September 2018. Nausea, vomiting, and loss of appetite lasted until shortly after the antibiotics were stopped in December 2019.

**Figure 1.**
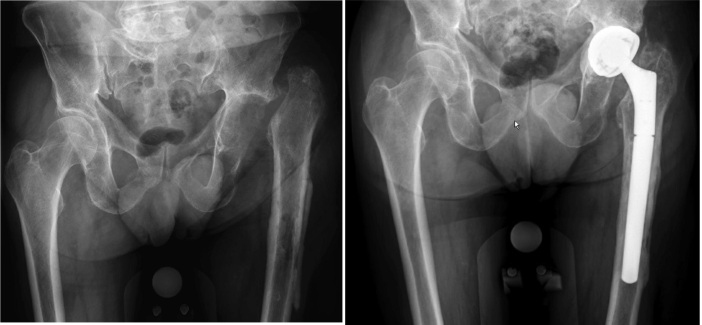
After explantation of total hip prosthesis and after final reimplantation of a new prosthesis.

The option of bacteriophage therapy was discussed and started on the patient’s own initiative with help from the Eliava Institute of Bacteriophage, Microbiology and Virology in Tbilisi, Georgia. He collected the formerly obtained tissue samples and sent them to Georgia. Susceptibility was retested for different antibiotics and phages. In the received documentation from Georgia, the *E. faecalis* appeared to have 4+ susceptibility, which was the maximum obtainable score, to 2 different types of bacteriophage: Pyophage and IntestiPhage. None of the tested antibiotics (Table 1) reached the same “sensitivity score” as that determined for these 2 phages. The *Enterococcus* even appeared to be resistant to doxycycline, which the patient was using as a suppressive regimen.

**Table 1.** List of tested bacteriophages and antibiotics

Bacteriophage sensitivity	
Fersis Phage	R
MonoStaph	R
Encophage	R
Pyophage	4+
IntestiPhage	4+
SES Phage	R
Antimicrobial sensitivity	
Amibac (amikacin)	R
Amoxicillin	2+
Ampicillin	1+
Ampiox (ampicillin)	3+
Aprid (ampicillin + sulbactam)	2+
Avelox (moxifloxacin)	3+
Biseptol (sulfamethoxazole + trimethoprim)	R
Ciprofloxacin	2+
Claforan (cefotaxime)	R
Clarithormycin	R
Dalacin	R
Doxycycline	R
Erythromycin	R
Floxan (levofloxacin)	2+
Fortum (ceftazidime)	R
Gentamicin	R
Meflocid (levofloxacin)	2+
Rifampicin	R
Sumamed (azithromycin)	R
Triaxon (ceftriaxon)	R
Zinnat (cefuroxime)	R

Our patient travelled to Georgia to retrieve his ampoules of Pyophages and IntestiPhages. They were delivered in 10-mL vials as an oral suspension. He started using them on June 13, 2018, and continued for 19 days. The Pyophages were taken in the morning and Intestiphages in the evening. After a 2-week pause, the bacteriophage therapy was restarted for another 19 days. Unfortunately, the exact composition of these phages and their concentration, expressed as plaque-forming units (PFUs), remain unknown to us and cannot be deduced from the patient leaflet. As recommended in the available literature, our patient received daily antibiotics during the 2 short periods of phage therapy. During the first period of phage therapy, he used oral amoxicillin 1,000 mg 4 times a day. During the second period, he used oral doxycycline 200 mg once a day. In December 2019, all antibiotics were stopped. He had no hip complaints when we saw him in our outpatient clinic in February 2021. No new cultures have been obtained up to the time of writing (July 2021). A time schedule of the treatment is presented in Figure 2.

**Figure 2.**
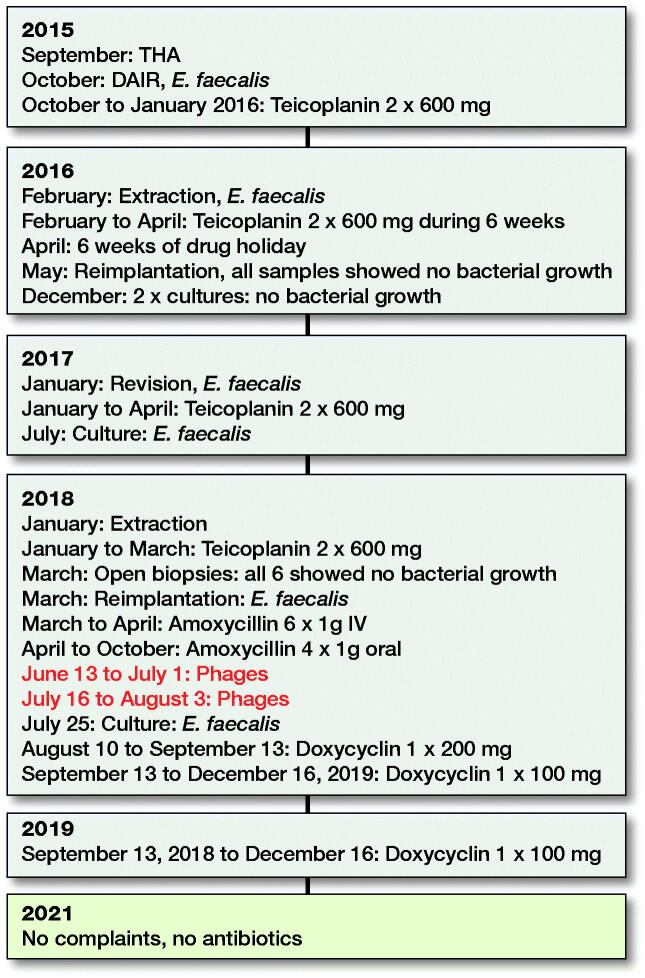
Time schedule of treatment.

## Discussion

*Enterococcus faecalis* is a microorganism known for its ability to develop biofilms. These biofilms play a key role in antibiotic resistance and recurrent periprosthetic joints infections (PJIs), which makes Enterococcus-induced PJIs particularly notorious. Besides a comprehensive antibiotic cure, 2-stage revision with delayed reimplantation remains the gold standard treatment for PJIs, as described by Renz et al. (2019). Both 2-stage revisions in our patient were performed following the global guidelines, as was the surgical procedure, including the removal of all cement residues and the duration of antibiotic treatment. The treatment of our patient involved a multidisciplinary approach, as recommended for PJI treatment, involving an infection specialist, a microbiologist, a pharmacist, and an orthopedic surgeon. By following an interdisciplinary standardized protocol, with successful extraction of the prosthesis and the surrounding cement, one should be able to eradicate 60–90% of PJIs (Akanda et al. 2018, Karczewski et al. 2019). In septic situations where a 1- or 2-stage revision is not an option, as in the case of multiresistant microorganisms, little bone stock, poor quality of the surrounding tissues, or more than 1 failed prior septic revision and/or severe comorbidity, less attractive treatment options must be considered, such as chronic suppressive antibiotics and/or definite extraction of the prosthesis or a Girdlestone procedure. When antibiotic and conventional surgical treatments fail, one option is bacteriophage therapy. Bacteriophages were discovered over a century ago, a decade before the first antibiotic. The United States and Western Europe focused on the further development of antimicrobial agents, whereas the former Soviet Union and Eastern Europe continued the use of phages. Due to the growing resistance to antibiotics, there is renewed interest in phage therapy. Nowadays, within the orthopedic department, they are mainly used in the treatment of multidrug-resistant bacterial infections (Lin et al. 2017, Akanda et al. 2018). Phage therapy is not currently approved in the Western Europe, at least not for human use. This is mainly because of the lack of literature, documentation (Lin et al. 2017), and a regulatory framework (Fauconnier 2019). In bacteriophage therapy, viruses (phages) are used to treat bacterial infections by infiltrating these bacteria and inducing lysis. Phages are non-living biological entities containing RNA or DNA. This means they are dependent on their host, the bacteria, for survival and reproduction. In general, there are 2 types of phages: temperate phages, which integrate their material into the bacterial genome, and lytic phages, which take over the whole replication mechanism of the bacteria to produce more phages. These lytic phages have evident antibacterial activity, in which lysis of the bacteria occurs through the production of proteins, called endolysins (Sulakvelidze et al. 2001, Yilmaz et al. 2013, Lin et al. 2017, Akanda et al. 2018). After bacterial lysis, the phages are released and attack additional bacteria, thereby enhancing the effect. This implies that the synthesis of phages will be higher with a denser bacterial concentration, similar to that within a biofilm (Chaudry et al. 2017). Phages are host specific because they bond to 1 or a small number of receptors on the cell wall by recognizing bacterial surface proteins. This implies that they will attack only 1 type or a small variety of different bacteria (Sulakvelidze et al. 2001, Yilmaz et al. 2013, Lin et al. 2017, Akanda et al. 2018). The great advantage is that phage-resistant bacteria remain susceptible to other phages of a similar target range (Sulakvelidze et al. 2001, Yilmaz et al. 2013, Chaudry et al. 2017). These new phages can be processed rapidly. By using several phages in combination, 1 can delay the development of resistance, as described by Sulakvelidze et al. (2001). Furthermore, phages can dissolve the bacterial biofilm (Ryan et al. 2012, Chaudry et al. 2017).

In the English language, publications on in vivo bacteriophage therapy as a treatment for bone-related infections are scarce. Available data has shown a synergetic effect of phage therapy in combination with antibiotics (Yilmaz et al. 2013, Akanda et al. 2018). In an in vitro model, the pre-antibiotic administration of phages appeared to be the most efficient approach (Chaudry et al. 2017, Akanda et al. 2018). It is believed that use of phages will lower the required antibiotic concentration (Ryan et al. 2012, Akanda et al. 2018). This is why our patient took his previously prescribed antibiotics and his phage cocktails simultaneously. None of the published phase I studies reported any adverse outcomes (Sulakvelidze et al. 2001, Ryan et al. 2012, Yilmaz et al. 2013, Chaudry et al. 2017, Lin et al. 2017, Akanda et al. 2018). Similarly, our patient did not perceive any relevant side effects.

The disease-causing bacterium must be identified before phage susceptibility can be determined and therapy can begin (Yilmaz et al. 2013). Therefore, phage therapy is not a viable treatment option for acute PJI. Another limitation is that published studies have only focused on the treatment of S. aureus- and Pseudomonas-induced PJIs (El Helou et al. 2008, Ryan et al. 2012, Yilmaz et al. 2013, Chaudry et al. 2017, Akanda et al. 2018, Renz et al. 2019).

In conclusion, to our knowledge, this is the first case report on bacteriophage therapy for an *E. faecalis* PJI. Due to increasing rates of resistance to currently used antibiotics or difficult-to-treat PJIs, alternative treatment options are of high importance. One of these “new” options is bacteriophage therapy, which is interesting due to its ability to attack biofilms associated with PJIs. The available literature reports excellent safety profiles and promising results for combination therapy with regular antibiotics. However, many aspects of phage therapy remain unclear.
